# Identification and characterization of a marine bacterium extract from *Mameliella* sp. M20D2D8 with antiviral effects against influenza A and B viruses

**DOI:** 10.1007/s00705-024-05979-8

**Published:** 2024-02-07

**Authors:** Hyo-Jin Kim, Jun-Gyu Park, Kyeong-Seo Moon, Su-Bin Jung, Yong Min Kwon, Nam Seon Kang, Jeong-Hyeon Kim, Sang-Jip Nam, Grace Choi, Yeong-Bin Baek, Sang-Ik Park

**Affiliations:** 1 Laboratory of Veterinary Pathology, College of Veterinary Medicine, Chonnam National University, Gwangju, 61186 Republic of Korea; 2 Laboratory of Veterinary Zoonotic Diseases, College of Veterinary Medicine, Chonnam National University, Gwangju, 61186 Republic of Korea; 3 College of Veterinary Medicine and BK21 FOUR Program, Chonnam National University, Gwangju, 61186 South Korea; 4 Department of Microbial Resources, National Marine Biodiversity Institute of Korea, 75, Jangsan-ro 101beon-gil, Seocheon-gun, Chungcheongnam-do 33662 Republic of Korea; 5 Department of Chemistry and Nanoscience, Ewha Womans University, Seoul, 03760 Republic of Korea

**Keywords:** *Mameliella* sp., Influenza virus, Marine extract, Broad-spectrum therapeutics, Apoptosis

## Abstract

Despite significant improvements in vaccines and chemotherapeutic drugs, pathogenic RNA viruses continue to have a profound impact on the global economy and pose a serious threat to animal and human health through emerging and re-emerging outbreaks of diseases. To overcome the challenge of viral adaptation and evolution, increased vigilance is required. Particularly, antiviral drugs derived from new, natural sources provide an attractive strategy for controlling problematic viral diseases. In this antiviral study, we discovered a previously unknown bacterium, *Mameliella* sp. M20D2D8, by conducting an antiviral screening of marine microorganisms. An extract from M20D2D8 exhibited antiviral activity with low cytotoxicity and was found to be effective *in vitro* against multiple influenza virus strains: A/PR8 (IC_50_ = 2.93 µg/mL, SI = 294.85), A/Phil82 (IC_50_ = 1.42 µg/mL, SI = 608.38), and B/Yamagata (IC_50_ = 1.59 µg/mL, SI = 543.33). The antiviral action was found to occur in the post-entry stages of viral replication and to suppress viral replication by inducing apoptosis in infected cells. Moreover, it efficiently suppressed viral genome replication, protein synthesis, and infectivity in MDCK and A549 cells. Our findings highlight the antiviral capabilities of a novel marine bacterium, which could potentially be useful in the development of drugs for controlling viral diseases.

**Supplementary Information:**

The online version contains supplementary material available at 10.1007/s00705-024-05979-8.

## Introduction

RNA viruses pose a serious threat to animal and human health due to emerging and re-emerging outbreaks of diseases and have a substantial impact on the global economy. Examples of pathogenic RNA viruses include influenza A virus (IAV), severe acute respiratory syndrome coronavirus 1 (SARS-CoV-1), and SARS-CoV-2 [1]. A major challenge presented by RNA viruses is the high degree of genetic diversity arising from from their high mutation rate and rapid adaptation [2, 3]. To address the issue of viral mutation, persistent and enhanced surveillance is necessary, along with innovations in antiviral therapy. Consequently, antiviral drugs, particularly those originating from natural sources with broad specificity, are needed to manage emerging virus variants.

Recently, natural products from marine sources have attracted attention because of the anti-inflammatory, antitumor, antimicrobial, antiviral, antimalarial, and antioxidant properties of bacterial components or secondary metabolites [2–8]. Annually, more than 1,200 novel natural products are discovered in marine organisms such as algae and microorganisms [6]. Natural compounds with antiviral potential have undergone various stages of clinical trials, and several of them are commercially available [6, 8]. For example, vidarabine, whose lead structure, spongouridine, was originally isolated from a marine sponge, is currently used for the treatment of herpes simplex virus (HSV) infection [9]. Bacterial exopolysaccharides (EPSs) have recently been found to have antiviral and anticancer effects and to be involved in host-microorganism interactions [10–12]. EPSs have been shown to stimulate type I interferon (IFN) production and expression of IFN-stimulated genes, enhancing host-innate immunity [13]. In particular, the IFN-dependent mediator TRAIL (TNF-related apoptosis-inducing ligand) has been found to limit the spread of influenza virus [14]. In addition, EPS from *Pediococcus*, *Leuconostoc*, and *Lactobacillus* bacteria have been shown to completely suppress the synthesis of infectious particles of human adenovirus by modulating the cell cycle [10]. Certain EPSs can cause aggregation of virus particles through their glucose and fucose moieties [15, 16].

Previous research has shown that the use of bacterial byproducts can be a promising approach for the treatment of viral diseases. In this study, a novel bacterium, *Mameliella* sp. M20D2D8 (accession number OR481697), was discovered by conducting comprehensive antiviral screening of marine microbes. This bacterium belongs to the genus *Mameliella* of the family *Roseobacteraceae* and was found to have 100% sequence identity to *Mameliella alba* JLT354-W^T^ (accession number: EU734592) in its 16s rRNA gene. The M20D2D8 extract exhibited antiviral activity with low cytotoxicity and was effective against IAV and influenza B virus (IBV) strains, exerting its effect in the post-entry stages of viral replication. Members of the genus *Mameliella* are a source of biologically active EPS [17]. Many studies have revealed that bacterial EPSs have antiviral activity, inhibiting virus reproduction in the late stages of infection and stimulating the immune system [18, 19]. We found that the M20D2D8 extract suppressed viral replication by enhancing apoptosis. The antiviral response efficiently suppressed viral genome replication, protein synthesis, and infectivity, resulting in successful protection of the host cells against multiple influenza viruses.

## Materials and methods

### Isolation of the bacterial strain and culture conditions

In July 2020, bacteria of the genera *Mameliella*, *Roseovarius*, *Sulfitobacter*, *Tritonibacter*, and *Thalassobius*, were isolated from a hypersaline water sample (salinity, 100 practical salinity units) collected from a solar saltern in Taean (Chungnam Province, KR). The water sample was spread on ZoBell medium (0.5 g peptone, 0.1 g yeast extract, and 0.001 g ferric phosphate [FePO_4_] per liter of 20% distilled water and 80% filtered seawater) using the standard dilution-plating method and incubated at 25°C for 5 days as described previously [20]. Isolated colonies were picked and transferred to fresh agar plates until they were pure. The purified strain was routinely cultured on marine agar (MA) 2216 (Difco) at 25°C and preserved in 20% (v/v) glycerol at -80°C. The bacterial isolate was deposited in the Microbial Marine Bio Bank (MMBB) of the National Marine Biodiversity Institute of Korea (MABIK) under the number MI00006275.

### Phylogeny of 16S rRNA gene sequences

Genomic DNA was extracted using an Exgene DNA extraction kit (Gene All, KR) according to the manufacturer’s instructions. Amplification of the 16S rRNA gene was performed by polymerase chain reaction (PCR) using the bacteria-specific universal primers, 27F and 1492R [21]. The amplified partial 16S rRNA gene was sequenced using an Applied Biosystems automated sequencer (ABI 3730XL) at Macrogen Co. Ltd. (Seoul, KR) and assembled using the Geneious program (version 9.0.5) to obtain a nearly full-length 16S rRNA gene sequence, which has been deposited in the GenBank database under the accession number OR481697. The phylogenetic position of strain the M20D2D8 strain was determined using the EzBioCloud server (ezbiocloud.net/identify) [22] by comparing the 16S rRNA gene sequence (1378 nucleotides) with other published sequences. Phylogenetic trees based on 1314 unambiguously aligned sequences were constructed using the neighbor-joining (NJ), maximum-likelihood (ML), and maximum-parsimony (MP) algorithms [23–25] in MEGA X [26]. To evaluate the robustness of the tree topologies, 1000 bootstrap performed for each of the three algorithms [27].

### Preparation of bacterial extracts

Bacterial extracts were prepared using a slight modification of the method described by Choi et al. [28]. Bacteria, including members of the genera *Mameliella*, *Roseovarius*, *Sulfitobacter*, *Tritonibacter*, and *Thalassobius*, were first cultured in 2.5-L Erlenmeyer flasks containing 1 L of marine broth (total, 5 L) under a 60 µmol m^− 2^ s^− 1^ LED light at 25℃. Subsequently, 20 L of medium in a panel or column-type photobioreactor was inoculated with the inoculum at a concentration of 10^4^ CFU mL^− 1^, and the cells were cultured under the same conditions for 10–20 days with shaking at 150 rpm. At the end of the culture period, the culture broth was extracted twice with an equal volume of ethyl acetate (EtOAc). The EtOAc-soluble component was then combined and dried using a vacuum evaporator. A crude total of 150 mg of bacterial extract was obtained from each species. For testing of antiviral activity, the extracts were dissolved in dimethyl sulfoxide (DMSO).

### Cell culture

Madin-Darby canine kidney (MDCK) or A549 cells were obtained from the American Type Culture Collection (ATCC) (Manassas, VA, USA) and cultured in Dulbecco’s modified Eagle’s medium (DMEM) (Welgene Inc., GS, KR) supplemented with 10% fetal bovine serum (FBS) (Thermo Fisher Scientific, Waltham, MA, USA) and 1% penicillin/streptomycin (Lonza, Basel, CH) as described elsewhere [29]. Subcultured MDCK cells were grown for 3–4 days in an incubator at 37°C in the presence of 5% CO_2_ until the next passage.

### Viruses

Influenza virus A/Puerto Rico/8/1934 H1N1 (A/PR8), influenza virus A/Philippines/2/82 H3N2 (A/Phil82), and influenza virus B/Yamagata/16/88 (B/Yamagata) were purchased from ATCC. IAV and IBV were subsequently grown in MDCK cells supplemented with DMEM containing 2 µg of tosyl phenylalanyl chloromethyl ketone (TPCK)-treated trypsin (Thermo Fisher Scientific) per mL until reaching a sufficient titer, as described elsewhere [30]. The stocks of each influenza virus strain were stored at -80℃ until use. Virus titration was done by the TCID_50_ method as described below [31].

### Median tissue culture infectious dose (TCID_50_)

The titers of the A/PR8, A/Phil82, and B/Yamagata stains were determined by the TCID_50_ method as described elsewhere [31]. In brief, a series of tenfold dilutions of each virus was prepared in DMEM with a final dilution of 10^7^-fold. Then, MDCK cells in a 96-well plate were inoculated with viral suspension supplemented with 2 µg of TPCK-treated trypsin per mL. After 3–4 days, the plate was washed with Dulbecco’s phosphate-buffered saline (DPBS) (Lonza, Basel, CH), and each well was fixed with 100 µL of 4% paraformaldehyde (PFA) (Sigma-Aldrich, Inc., St. Louis, MO, USA). After 10 min of fixation at room temperature (RT), 100 µL of 1x crystal violet solution (Duksan Pure Chemicals, Ansan, KR) was added to each well, and the intact cells were stained for 10 min at RT. The TCID_50_ was calculated by the Spearman–Karber method [32]. Additionally, the progeny viruses were titrated by the TCID_50_ method, using the supernatant of virus-infected cells treated with a vehicle or the above extracts at 5 days postinfection (dpi) as described above.

### Cytotoxicity assay

Cytotoxicity was evaluated using the water-soluble tetrazolium salt (WST) assay with slight modifications [33]. Five marine organism extracts were prepared in triplicate in a 96-well plate at the following concentrations: 1000, 500, 250, 100, 50, 25, 10, 5, 2, 1, 0.1, and 0.01 µg/mL. MDCK cells in another 96-well plate were treated with the diluted extract and incubated for 2 days at 37℃. Afterward, the medium was exchanged with diluted Cellvia solution (GW Vitek, Seoul, KR) according to the manufacturer’s instructions. After 30 min of incubation at 37°C, the absorbance was measured by an ELISA microplate reader (Thermo Fisher Scientific) at 450 nm with a reference absorbance at 650 nm. The output values were normalized and calculated as the half-maximal cytotoxic concentration (CC_50_) with a generation of a dose-response curve.

### Antiviral screening of extracts by pre- and post-infection treatment

Antiviral screening was carried out by measuring inhibition of the viral cytopathic effect (CPE) as described previously [34]. Briefly, marine organism extracts were prepared as described previously, in triplicate, in a 96-well plate at the following concentrations: 1000, 500, 250, 100, 50, 25, 10, 5, 2, 1, 0.1, and 0.01 µg/mL. Before viral infection, MDCK cells in a 96-well plate were treated with the diluted extract for 1 h at 37°C. Then, the cells were infected with influenza virus A/PR8 (multiplicity of infection [MOI] = 0.04), A/H3N2 (MOI = 0.01), or B/Yamagata (MOI = 0.1) for 1 h at 37°C. The plate was washed with DMEM, and the cells were treated with the serially diluted extracts supplemented with 2 µg of TPCK-treated trypsin per mL. After 48 h at 37°C, the plate was washed with DPBS and treated with 0.5 mg of soluble MTT (3-(4,5-dimethylthiazol-2-yl)-2,5-diphenyltetrazolium bromide) (Sigma-Aldrich) reagent per ml (200 µL/well) for 4 h at 37°C in a 5% CO_2_ incubator. After incubation with DMSO for 10 min on a shaker, the colorimetric change of each well was measured using an ELISA microplate reader by reading the optical density at 570 nm. The data were used to calculate the half-maximal inhibitory concentration (IC_50_) and CC_50_, using GraphPad Prism (version 9.5.1). The selectivity index (SI) was calculated using sigmoidal dose-response curves (GraphPad Software, CA, United States) based on the following equation: SI = mean CC_50_ / mean IC_50_, where CC_50_/IC_50_ is the ratio of CC_50_ to IC_50_.

### Selection of pre-, co-, or post-treatment of M20D2D8 extract

Based on pre- and post-treatment assays, one extract that showed the strongest effect was selected. MDCK cells in a 96-well plate were treated in triplicate with the chosen extract at different concentrations in serial dilutions ranging from 0.01 to 100 µL/mL. The inoculation dose of influenza viruses was the same as before (at an MOI of 0.04 FFU/well), while the timing of inoculation was adjusted for pre-, co-, and post-infection treatment as described previously [35]. Briefly, for pre-treatment, the extracts were added 1 h before viral infection at 37°C. For co-treatment, different concentrations of the extracts prepared in different concentrations were mixed with viruses for 1 h at 37°C and transferred to the cells. Post-treatment of the extracts was performed after viral absorption. In each procedure, the maintenance medium was supplemented with 2 µg of TPCK-treated trypsin per mL, and the cell pellet and supernatant were collected after incubation for 48 h at 37°C. The antiviral activity was then measured in the same manner as in the previous screening.

### Real-time quantitative reverse transcription polymerase chain reaction (RT-qPCR)

Total RNA was extracted using a QIAamp Viral RNA Mini Kit (QIAGEN, Venlo, The Netherlands) according to the manufacturer’s instructions as described previously [36]. Subsequently, cDNA synthesis was performed using total RNA and a SensiFAST™ SYBR Lo-ROX One Step Kit (Meridian Bioscience, OH, USA). RT-qPCR was carried out as described previously [37]. The master mix, comprising 10 µl of 2× SensiFAST™ SYBR Lo-ROX One Step Mix, 0.8 µL of 10 µM forward and reverse primers, 0.2 µL of reverse transcriptase, 0.4 µL of Ribosafe RNase inhibitor, 5.8 of µL water, and 2 µL of RNA template, had a total volume of 20 µL. The primers IAV PB1-F (F:5'-GGCCCTTCAGTTGTTCATC-3') and IAV PB1-R (3'-GTGTAAGGACTTCAGACG-5') were used in the reaction. The reaction was performed using a LineGene 9600 Plus Real-time PCR detection system (Bioer technology, Hangzhou, CN).

### Immunofluorescence assay (IFA)

In correspondence with post-treatment experiments, MDCK cells in an 8-well chamber were infected with A/PR8 at an MOI of 0.04 or mock-infected and then treated with the vehicle or extract. At 24 h postinfection, the cells were washed and fixed with 4% PFA (Thermo Fisher Scientific) for 10 min at RT. The cells were permeabilized with 0.2% Triton X-100 (Thermo Fisher Scientific) for 10 min at RT, and 5% bovine serum albumin (BSA) (GenDEPOT, Texas, USA) in DPBS was added to block nonspecific reactions. The cells were then incubated overnight at 4°C with primary antibody against IAV nucleoprotein (NP) (Abcam, Cambridge, UK), followed by incubation with a secondary antibody against mouse IgG conjugated with Alexa Fluor (AF) 488 for 1 h at RT. After nuclear staining with a 1 µg/mL DAPI solution for 10 min at RT, the slide was prepared for observation by confocal microscopy.

### Flow cytometry

MDCK cells were grown to 90% confluence on a 6-well plate. The cells were then inoculated with influenza virus A/PR8 at an MOI of 1. After 1 hour, 50 µg of M20D2D8 extract per mL and the positive control, chloroquine, were applied, and the cells were incubated at 37℃ until 12 h postinfection. The cells were then collected by treatment with 1x trypsin for 5 min and centrifugation at 3000 rpm for 3 min in a microcentrifuge (Hanil, Gimpo, KR). After permeabilization with 0.2% Triton X-100 at RT for 5 min, the cells were blocked with 5% BSA for 1 hour at RT. Then, anti-IAV NP antibody was applied and an In Situ Cell Death Detection Kit (Roche, Basel, Switzerland) was used to assess cell viability. Flow cytometry was performed using an Attune NxT Flow System (Thermo Fisher Scientific).

### Virus attachment and penetration assay

As described previously [38], attachment and penetration assays were conducted with 5 × 10^5^ MDCK cells in a 24-well plate. In the case of the attachment assay, the cells were treated with M20D2D8 extract at 50 µg/mL and 100 µg/mL for 30 min at 4 ℃. Then, strain A/PR8 was added to the cells at an MOI of 1, and the cells were kept at 4 ℃ for 1 h. After thorough washing, the cells were incubated at 37℃ for 20 h and viral RNA was detected by RT-qPCR.

In the case of the penetration assay, MDCK cells were cooled to 4℃, strain A/PR8 was added at an MOI of 1, and the cells were kept for 1 h at 4℃. After thorough washing, M20D2D8 extract was applied to the cells at 37℃ for 10 min. After washing with Tris buffer (pH 3.0), the cells were incubated for 20 h at 37℃. Viral RNA was detected by RT-qPCR. As positive controls, antibodies against IAV HA and chloroquine were used for the attachment and penetration assay, respectively.

## Results

### Isolation of the bacterial strain and phylogenetic analysis

**Fig. 1:**
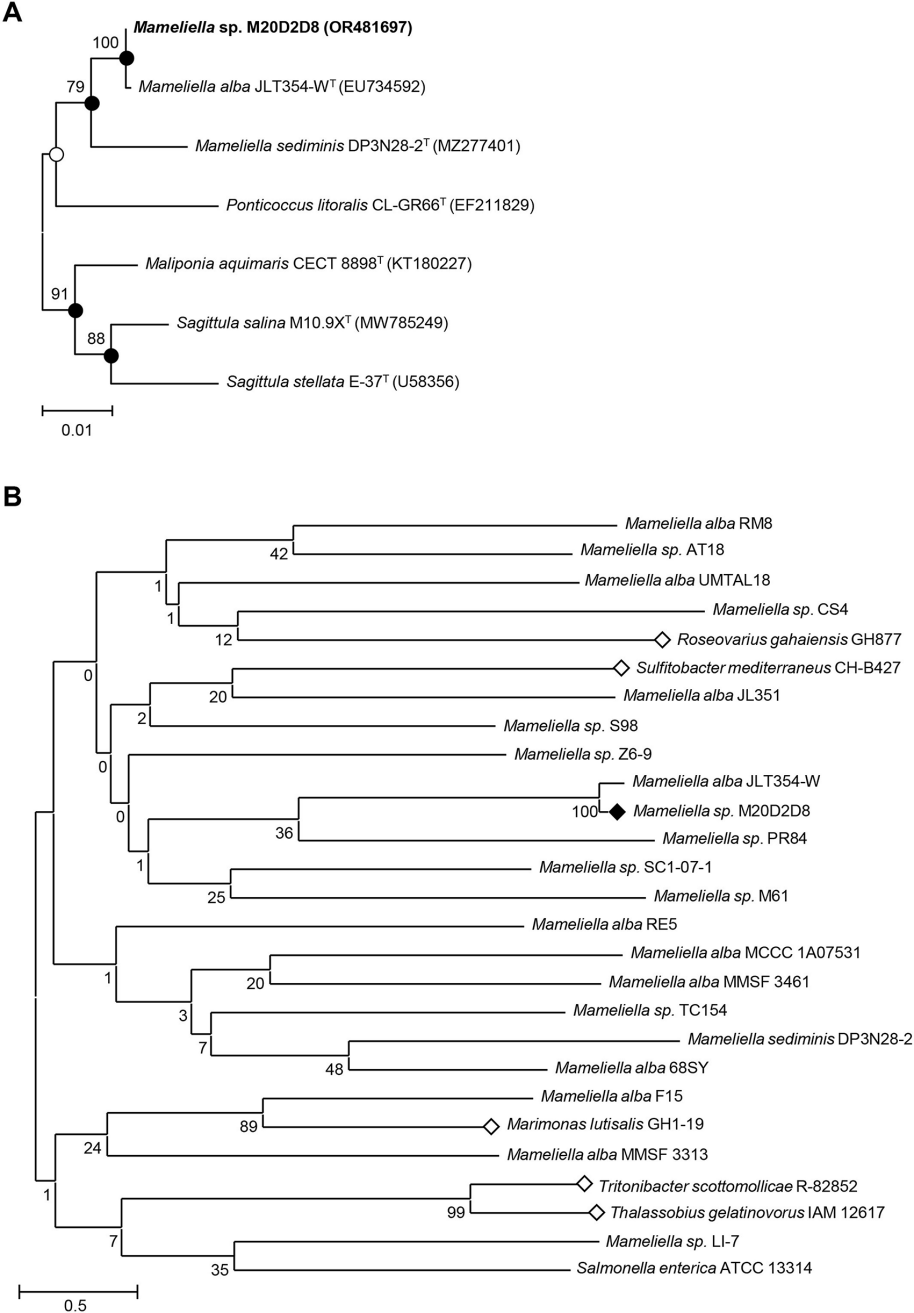
Phylogenetic analysis and antiviral screening of bacterial extracts. (**A**) A neighbor-joining tree based on 16S rRNA gene sequences, showing the phylogenetic relationships of strain M20D2D8 (in bold type) and closely related bacteria. GenBank accession numbers are shown in parentheses. Bootstrap values above 70% are shown at nodes as percentages out of 1000 replicates. Closed and open circles indicate nodes that were obtained using three methods (neighbor-joining, maximum-likelihood, and maximum-parsimony) or two methods, respectively. Bar, 0.01 changes per nucleotide position. (**B**) Marine bacteria whose extracts were subjected to preliminary antiviral screening. Bar, 0.50 changes per nucleotide position

Sequence comparisons showed that the 16S rRNA gene sequence of strain M20D2D8 was 100% identical to that of *Mameliella* alba JLT354-W^T^, 98.16% identical to that of *M. sediminis* DP3N28-2^T^, and < 97.88% identical to those of other strains. Phylogenetic analysis using the NJ, ML, and MP algorithms revealed that strain M20D2D8 formed a phylogenetic lineage with *M. alba* JLT354-W^T^ and *M. sediminis* DP3N28-2^T^ within the genus *Mameliella* (Fig. 1A).

### Antiviral screening of *Mameliella* sp. M20D2D8

**Fig. 2:**
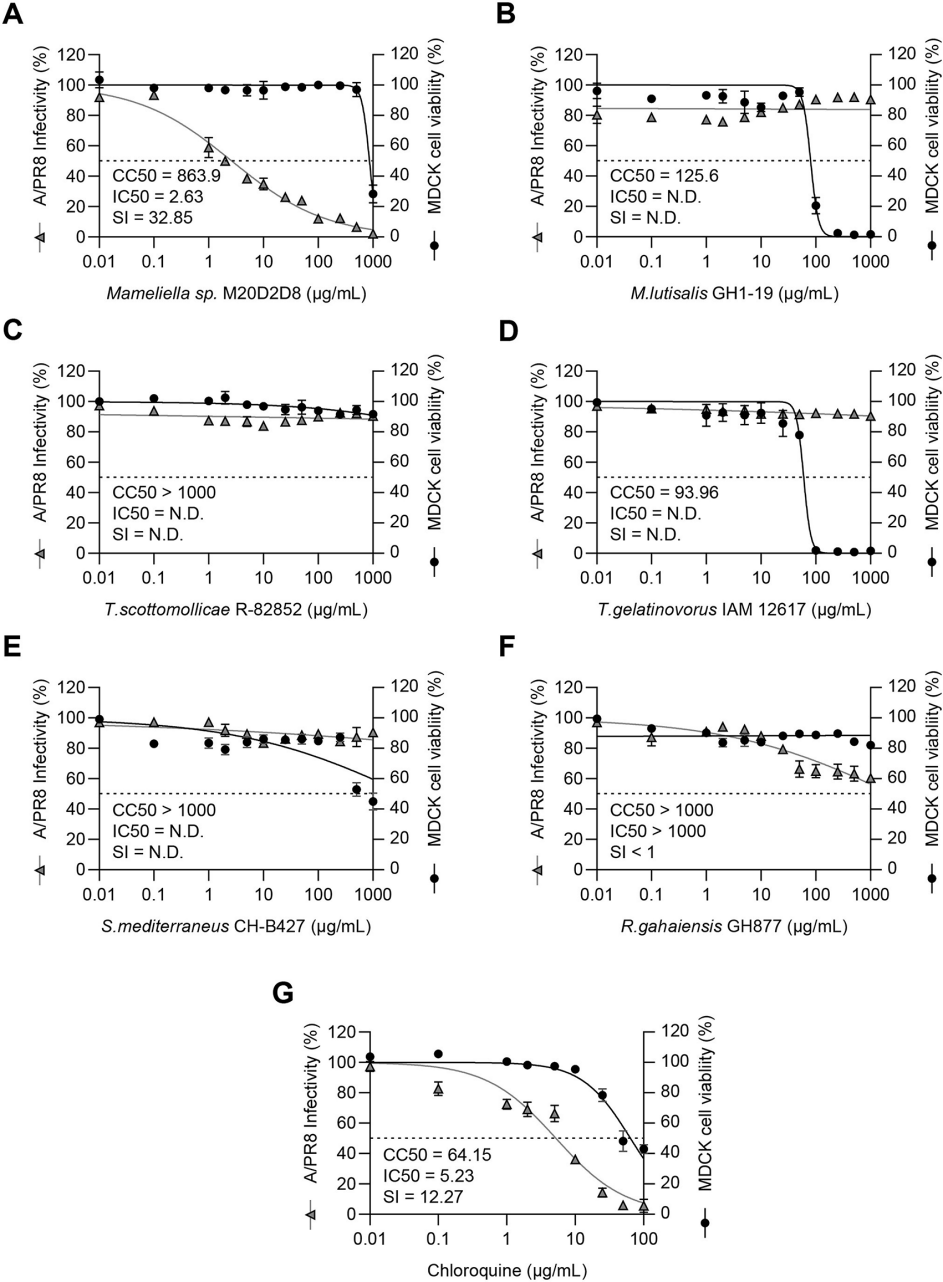
Antiviral screening of extracts of six marine bacteria by pre- and post-infection treatment. Antiviral activity was measured as the inhibitory effect on the cytopathic effect (CPE) induced in MDCK cells by influenza virus A/PR8 (MOI = 0.04). CC_50_ (µg/mL), IC_50_ (µg/mL), and SI values were calculated using GraphPad Prism 9.5.1. (**A**) *Mameliella* sp. M20D2D8, (**B**) *M. lutisalis* (strain GH1-19), (**C**) *Tritonibacter scottomollicae* (strain R-82852), (**D**) *Thalassobius gelatinovorus* (strain IAM 12617), (**E**) *Symphodus mediterraneus* (strain CH-B427), (**F**) *Roseovarius gahaiensis* (strain GH877), (**G**) chloroquine

We examined the antiviral activity of extracts of strain M20D208 and other closely related bacterial strains by examining the inhibitory effect on CPE induced by influenza virus A/Puerto Rico/8/1934(H1N1) (A/PR8) (Fig. 1B, Fig. 2). Of these strains, strain M20D2D8 exhibited the highest SI (CC_50_ = 863.9 µg/mL, IC_50_ = 2.63 µg/mL, SI = 32.85). Moreover, the extract from strain M20D2D8 showed stronger antiviral activity than chloroquine (CC_50_ = 64.15 µg/mL, IC_50_ = 5.23 µg/mL, SI = 12.27), an FDA-approved antiviral drug.

### Minimal effect of M20D2D8 on viral entry

To investigate which stages of the influenza virus life cycle are affected by the M20D2D8 extract, the extract was applied to cells before (pre-treatment), during (co-treatment), and after (post-treatment) infection with influenza virus strain A/PR8. To examine the pre-treatment effect, MDCK cells were treated with the extract at various concentrations and then infected with the virus at an MOI of 0.04. At 48 h postinfection, the viral infectivity was examined (Supplementary Fig. S1A), but no clear antiviral effect was observed at any concentration of the extract (Supplementary Fig. S1B and C). To examine the co-treatment effect, the M20D2D8 extract was mixed with the virus at an MOI of 0.04, and the mixture was then transferred to MDCK cells (Supplementary Fig. S1D). Again, no antiviral effect was observed (Supplementary Fig. S1E), and there was no reduction in the number of viral genome copies (Supplementary Fig. S1F).

The effect of the bacterial extract on viral entry was further examined using viral attachment and penetration assays performed in MDCK cells inoculated with the virus at an MOI of 1 (Supplementary Fig. S2). In the attachment assay, RT-qPCR revealed that pre-treatment with the extract affected viral binding to the host cell (Supplementary Fig. S2A), and the assay was validated using an antibody against the H1N1 HA protein. A penetration assay showed that the number of viral genome copies decreased significantly when the extract was added during the post-attachment step in (Supplementary Fig. S2B), and the assay was validated using chloroquine, an inhibitor of endosome trafficking. The inconsistency between the viral attachment and penetration data and the infectivity measurements might have been due to a residual effect of the M20D2D8 extract in the host cell, given that no inhibition was found in CPE and viral genome copies after a longer incubation time (Supplementary Fig. S1).

### Post-treatment antiviral effect of M20D2D8 extract due to increased apoptosis

**Fig. 3:**
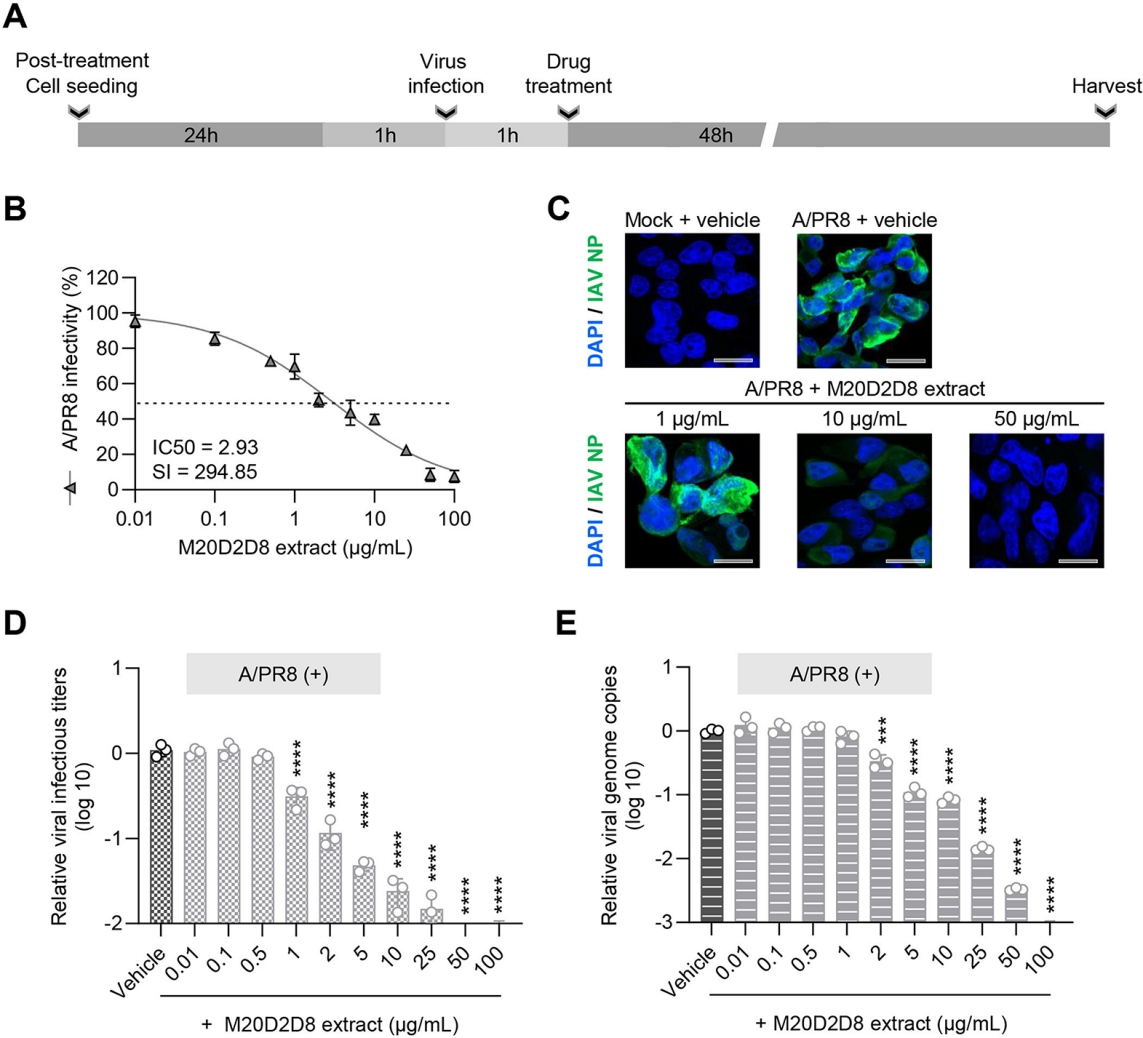
Antiviral effect of M20D2D8 extract applied to cells after infection with influenza virus A/PR8 (MOI = 0.04). (**A**) Schematic diagram of the experimental procedure. (**B**) IC_50_ and SI of M20D2D8 extract measured by CPE inhibition assay. (**C**) Viral protein synthesis detected by IFA. (**D**) Determination of the number of viral genome copies by RT-qPCR. (E) Progeny virus production measured by TCID_50_. All data are presented as the arithmetic mean ± S.D. from three independent experiments. *, *P* < 0.05; **, *P* < 0.01; ***, *P* < 0.001, ****, *P* < 0.0001

We investigated the antiviral potential of M20D2D8 extract in the post-entry stages of viral replication (Fig. 3). Briefly, MDCK cells were infected with influenza virus strain A/PR8 at an MOI of 0.04 and treated with the extract at various concentrations (0.01–100 µg/mL) (Fig. 3A). The results showed highly significant inhibition of CPE induced by IAV infection in a dose-dependent manner, with a much greater antiviral effect (IC_50_ = 2.93 µg/mL, SI = 294.85) than that of chloroquine (IC_50_ = 5.23 µg/mL, SI = 12.27) (Fig. 3B). Treatment with M20D2D8 extract resulted in a gradual decrease in viral protein synthesis, genome copies, and infectivity with increasing concentration of the inhibitor, and this effect was statistically significant (Fig. 3C-E). Thus, the antiviral effect occurred after of viral entry.

**Fig. 4:**
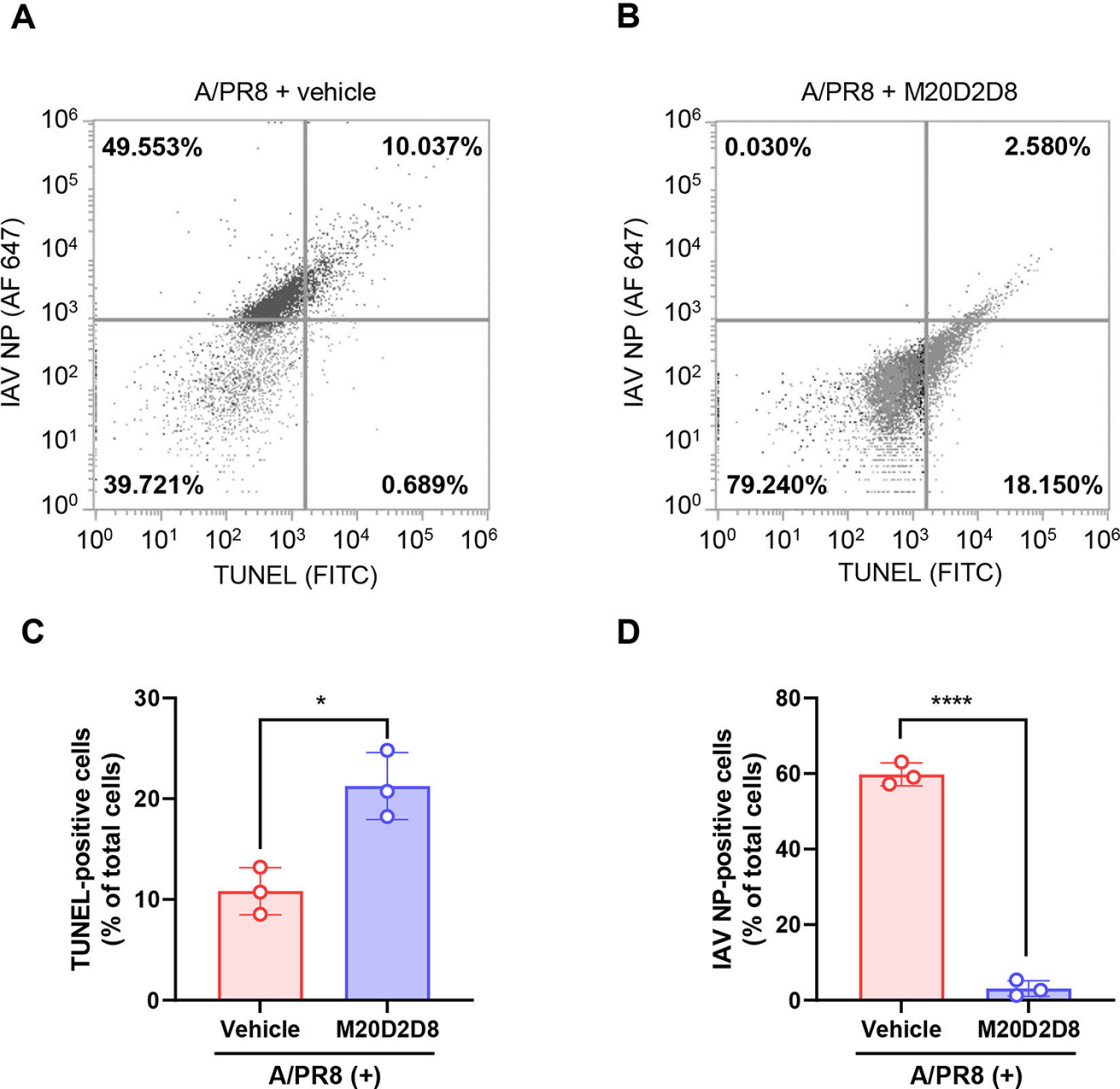
(**A**-**B**) Flow cytometry analysis of apoptosis after infection with influenza virus A/PR8 (MOI = 1). The levels of apoptosis and viral proteins were measured in dot plots using a TUNEL assay and antibody against IAV NP, respectively. (**A**) A/PR8-infected, vehicle-treated cells. (**B**) A/PR8-infected, M20D2D8-extract-treated cells. (**C**-**D**) Summary of flow cytometry data. Quantification of TUNEL- (**C**) and virus-positive cells (**D**) treated with the vehicle or M20D2D8 extract. All data in the graphs are presented as the arithmetic mean ± S.D. from three independent experiments. *, *P* < 0.05; ****, *P* < 0.0001

The effect of the M20D2D8 extract on apoptosis and viral replication was evaluated by flow cytometry (Fig. 4), and apoptotic DNA fragments and viral proteins were detected using a terminal deoxynucleotidyl transferase dUTP nick end labeling (TUNEL) assay and an antibody against IAV NP, respectively (Fig. 4A and B). The results showed a significant increase in apoptosis after treatment with the M20D2D8 extract (Fig. 4C). Treatment with the M20D2D8 extract almost completely eliminated expression of viral proteins in A/PR8-infected cells (Fig. 4D). These data show that the bacterial extract efficiently suppressed virus replication and induced apoptosis of infected cells.

### Antiviral activity of M20D2D8 extract against multiple influenza viruses

**Fig. 5:**
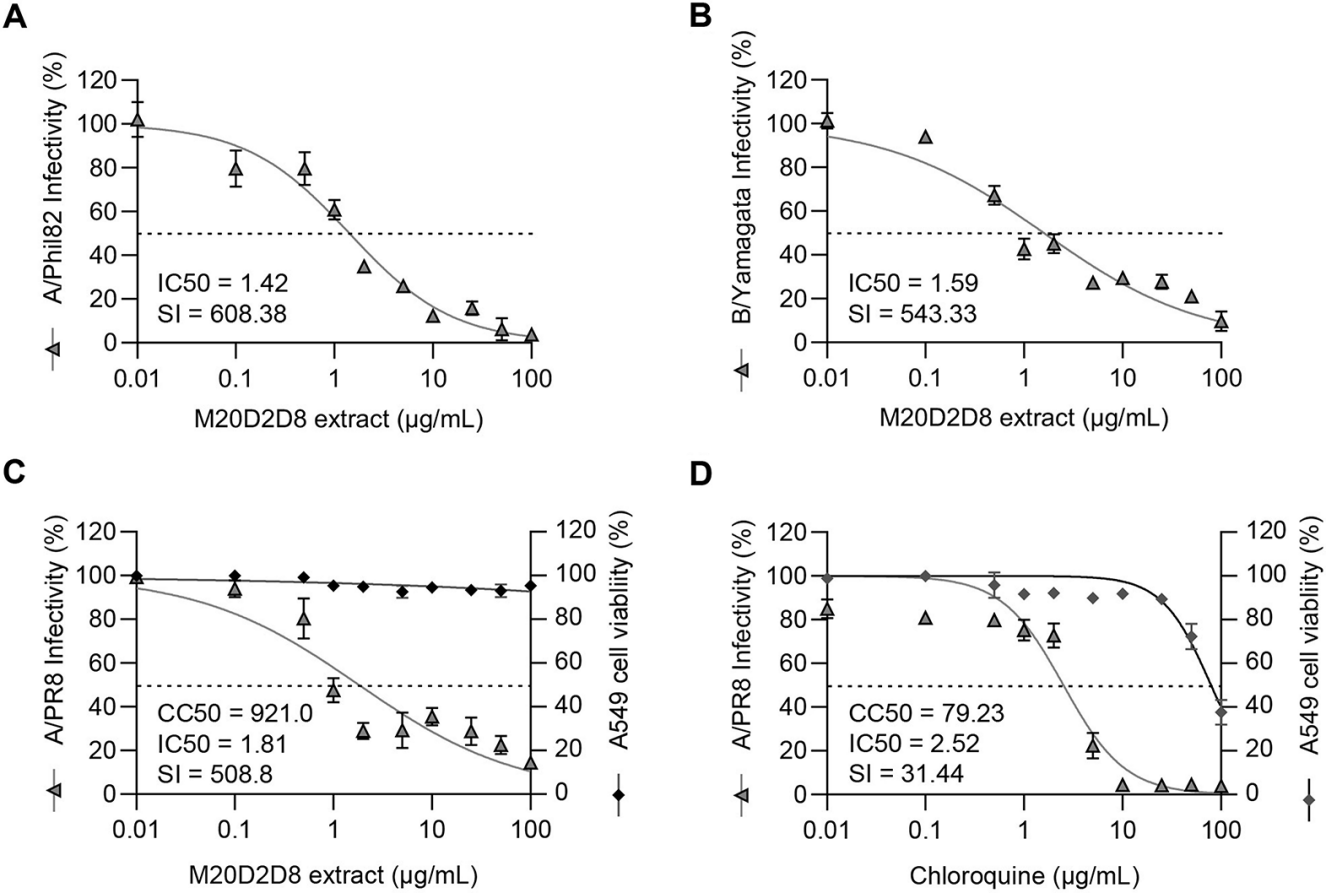
(**A**-**B**) Antiviral activity against multiple influenza viruses by post-infection treatment with the M20D2D8 extract. (**A**) strain A/Phil82. (**B**) strain B/Yamagata. (**C**-**D**) Antiviral activity of M20D2D8 extract (**C**) and chloroquine (**D**) in A549 cells

To examine the antiviral effect of the bacterial extract on other influenza viruses, an antiviral assay was carried out by measuring inhibition of CPE induced by influenza virus strains A/Phil82 and B/Yamagata as described above (Fig. 5). Post-treatment with the extract was found to inhibit both viruses (Fig. 5A and B). The IC_50_ and SI values against A/Phil82 were 1.42 µg/mL and 608.38, respectively, and those against B/Yamagata were 1.59 µg/mL and 543.33, respectively.

To test the antiviral effect in another cell line from a different organ, A549 cells were infected with strain A/PR8 at an MOI of 0.04 (Fig. 5C and D). The results showed that the M20D2D8 extract had a stronger antiviral effect (CC_50_ = 921.0 µg/mL, IC_50_ = 1.81 µg/mL, SI = 508.8) than chloroquine (CC_50_ = 79.23 µg/mL, IC_50_ = 2.52 µg/mL, SI = 31.44). Thus, the M20D2D8 extract exhibits a broad antiviral effect against multiple strains of influenza virus in different host cells, suggesting its therapeutic potential.

## Discussion

Emerging and reemerging infectious RNA viruses have a severe impact on the economy and human health. However, the available treatment options are very limited, since the effectiveness of the majority of antiviral drugs and vaccines can be greatly reduced by drug resistance or evasion of antibody-mediated host immunity [39]. Therefore, the development of new antiviral agents is urgently needed. Marine microorganisms, in particular, are potential sources of new drugs [40]. Previous studies have shown that bacterial have shown antiviral, anticancer, and antioxidant activities and can affect cellular immunity [41].

Preliminary experiments have shown that *Mameliella* sp. M20D2D8 extract has antiviral activity against hepatitis A virus (HAV) and porcine epidemic diarrhea virus (PEDV) (data not shown). Notably, *Mameliella* sp. is known to produce extremely large amounts of EPSs [17]. *In vitro* antiviral screening showed that M20D2D8 extract had a stronger antiviral effect against A/PR8 infection (IC_50_ = 2.63 µg/mL, SI = 32.85) than chloroquine (IC_50_ = 5.23 µg/mL, SI = 12.27), an FDA-approved antiviral drug. Based on previous pharmaceutical studies on natural products, these values indicate a potent antiviral effect [42].

Antiviral agents can affect different stages of the viral life cycle [43]. Understanding the pharmacological mechanisms involved is important for the development of new drugs [44, 45]. Here, we found that pre- or co-treatment with the M20D2D8 extract did not suppress IAV infection, although viral attachment and penetration assays suggested a possible antiviral effect, this effect was diminished when a higher MOI was used or the incubation period was lengthened. In contrast, post-treatment with the extract resulted in strong anti-IAV activity (IC_50_ = 2.93 µg/mL, SI = 294.85), suppressing viral genome replication, protein synthesis, and infectivity with a stronger effect than chloroquine. This suggests that the drug can be used for post-treatment therapy after viral infection.

Bacterial EPSs have been shown to exert antiviral activity by inhibiting the late stages of viral reproduction, reducing the infectivity of virions, and stimulating the immune system [18, 19]. In cells infected with influenza virus, EPS can stimulate type I IFN production and induce the expression of IFN-stimulated genes [13]. In addition, many compounds can promote apoptosis, thereby limiting the production of progeny viruses and re-infection, whereas cell death due to viral infection itself more commonly occurs through necroptosis or pyroptosis [46–48]. In our study, the M20D2D8 extract suppressed viral replication by inducing apoptosis. In order to identify the compound in the M20D2D8 extract that modulates apoptosis, further studies using liquid chromatography-tandem mass spectrometry (LC-MS/MS) will be required.

Our data demonstrated that the M20D2D8 extract exhibited strong antiviral activity against influenza virus A/Phil82 (IC_50_ = 1.42 µg/mL, SI = 608.38) and B/Yamagata (IC_50_ = 1.59 µg/mL, SI = 543.33). Moreover, its effectiveness was confirmed using human-origin cells. Although further studies using primary human respiratory epithelial cells will be required to assess the feasibility of its clinical use as a therapeutic drug, the M20D2D8 extract showed strong antiviral efficacy in a lung epithelial cell line (A549) against A/PR8 infection (CC_50_ = 921.0 µg/mL, IC_50_ = 1.81 µg/mL, SI = 508.8).

Toxicity is a limiting factor in the therapeutic application of many drugs with known antiviral activity [49, 50]. The current study showed that the bacterial extract potently blocks the replication of multiple influenza virus strains at low concentrations and has an SI value exceeding those of some FDA-approved drugs [51], suggesting its safety and effectiveness as a therapeutic agent. Recently, advances in technology have made feasible the cost-effective large-scale production of compounds obtained from marine microorganisms, potentially facilitating their application as pharmaceutical agents [52]. Several of them have been approved as antiviral agents for clinical use or testing in clinical trials, in particular for the treatment of human immunodeficiency virus (HIV) and HSV infections [53].

## Conclusion

We identified a novel bacterium, *Mameliella* sp. M20D2D8, through comprehensive antiviral screening of marine microbes. An extract from this bacterium exhibited strong antiviral activity *in vitro* with low cytotoxicity and activity against both IAV and IBV strains. It was found to primarily affect the post-entry stages of viral replication and to suppress viral replication by inducing apoptosis, resulting in decreased viral genome replication, protein synthesis, and infectivity. The discovery of the antiviral potential of this novel marine bacterium might play a role in future drug development and aid in controlling viral diseases.

### Electronic Supplementary Material

Below is the link to the electronic supplementary material

**Supplementary Material 1**: Fig. S1 includes antiviral effect of M20D2D8 extract by pre treatment and co-treatment against A/PR8 strain. Fig. S2 includes virus attachment and penetration assays.

## Data Availability

The data generated and/or analyzed in the current study are available from the corresponding author upon reasonable request.
