# Supplementary material for: Identification and characterization of a marine bacterium extract from Mameliella sp. M20D2D8 with antiviral effects against influenza A and B viruses
Source: Arch Virol. 2024 Feb 7;169(3):41. doi: 10.1007/s00705-024-05979-8 (PMC10850258; doi:10.1007/s00705-024-05979-8)
Supplement: Supplementary file 1 — Supplementary Material 1: Fig. S1 includes antiviral effect of M20D2D8 extract by pre treatment and co-treatment against A/PR8 strain. Fig. S2 includes virus attachment and penetration assays. [file 705_2024_5979_MOESM1_ESM.pdf]

Archives of Virology

Supplementary Materials for

Identification and characterization of a marine bacterium extract from *Mameliella* sp. M20D2D8 with antiviral effects against influenza A and B viruses

Hyo-Jin Kim[1*], Jun-Gyu Park[2*], Kyeong-Seo Moon[3], Su-Bin Jung[3], Yong Min Kwon[4], Nam Seon Kang[4], Jeong-Hyeon Kim[5], Sang-Jip Nam[5], Grace Choi[4†], Yeong-Bin Baek[1†], Sang-Ik Park[3†]

[1] Laboratory of Veterinary Pathology, College of Veterinary Medicine, Chonnam National University, Gwangju 61186, Republic of Korea

[2] Laboratory of Veterinary Zoonotic Diseases, College of Veterinary Medicine, Chonnam National University, Gwangju 61186, Republic of Korea

[3]Laboratory of Veterinary Pathology, College of Veterinary Medicine, Chonnam National University, Gwangju 61186, Republic of Korea; College of Veterinary Medicine and BK21 FOUR Program, Chonnam National University, Gwangju, 61186, South Korea.

[4] National Marine Biodiversity Institute of Korea, 75, Jangsan-ro 101beon-gil, Seocheon-gun, Chungcheongnam-do, 33662, Republic of Korea

[5]Department of Chemistry and Nanoscience, Ewha Womans University, Seoul 03760, Republic of Korea

[†]Correspondence to: gchoi@mabik.re.kr (G. Choi); ybbaek@jnu.ac.kr (Y.-B. Baek); sipark@jnu.ac.kr (S.-I. Park)

This PDF file includes:

Figures S1 to S2

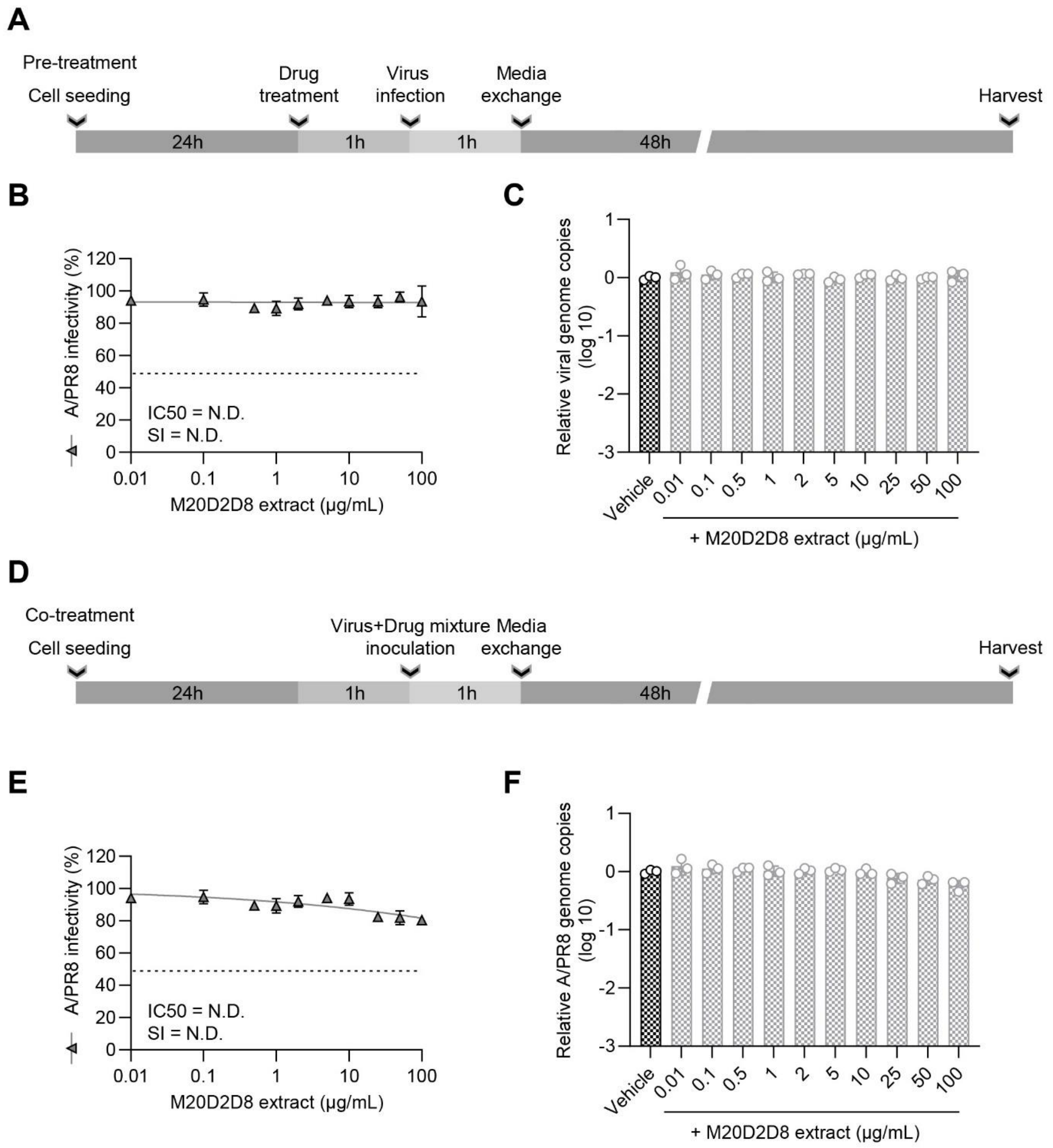

Supplementary Fig. S1 Antiviral effect of M20D2D8 extract by pre-treatment (**A-C**) and co-treatment (**D-F**) against A/PR8 strain. (**A**) Schematic diagram of virus inoculation and extract treatment. (**B**) IC_{50} and SI of M20D2D8 extract measured by CPE-inhibition assay. (**C**) Viral genome copies detected by RT-qPCR. (**D**) Schematic diagram of virus inoculation and extract treatment. (**E**) IC_{50} and SI of M20D2D8 extract measured by CPE-inhibition assay. (**F**) Viral genome copies detected by RT-qPCR. All data in the graphs are presented as arithmetic means ± S.D. from 3 independent experiments.

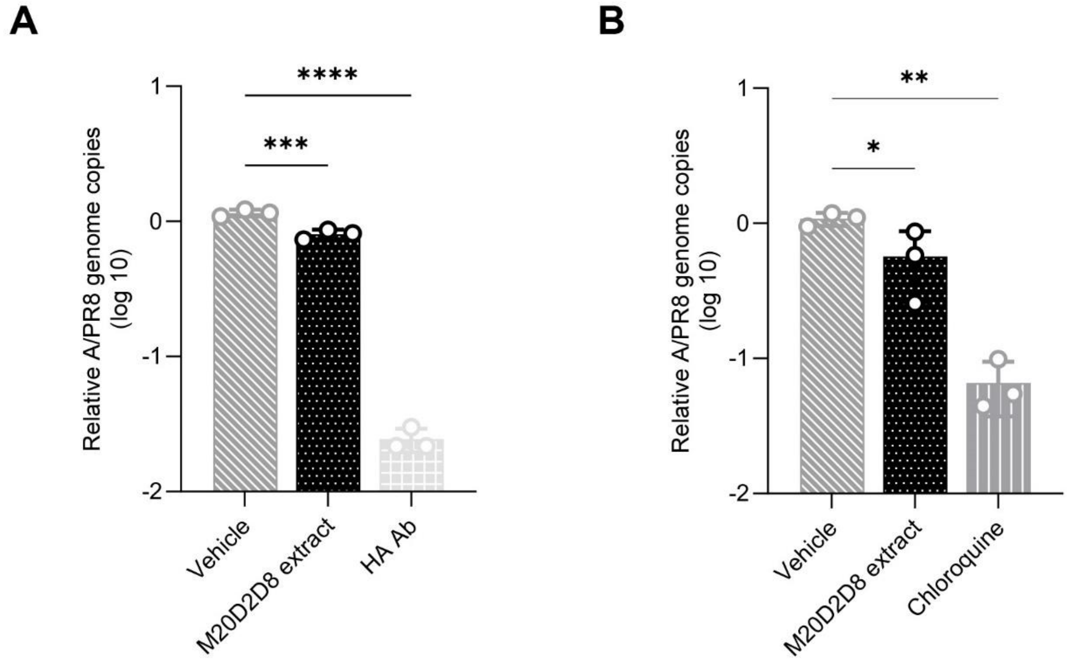

Supplementary Fig. S2 Virus attachment (**A**) and penetration assays (**B**). (**A**) Viral genome copies by pre-treatment of the extract before virus binding. (**B**) Viral genome copies by post-treatment after virus binding. All data in the graphs are presented as arithmetic means ± S.D. from 3 independent experiments. HA, haemagglutinin; IAV NP, nucleoprotein. *, *P*<0.05; **, *P*<0.01; ***, *P*<0.001, ****, *P*<0.0001.
